# Regulation and mechanism of action of miRNAs on insulin resistance in skeletal muscles

**DOI:** 10.1016/j.ncrna.2023.02.005

**Published:** 2023-02-16

**Authors:** Aferin Beilerli, Valentin Kudriashov, Albert Sufianov, Andrey Kostin, Sema Begliarzade, Tatiana Ilyasova, Yanchao Liang, Albert Mukhamedzyanov, Ozal Beylerli

**Affiliations:** a Department of Obstetrics and Gynecology, Tyumen State Medical University, 54 Odesskaya Street, 625023, Tyumen, Russia; b Gastric Cancer Center, West China Hospital of Sichuan University, China; c Educational and Scientific Institute of Neurosurgery, Рeoples’ Friendship University of Russia (RUDN University), Moscow, Russia; d Department of Neurosurgery, Sechenov First Moscow State Medical University (Sechenov University), Moscow, Russia; e Research and Educational Resource Center for Immunophenotyping, Digital Spatial Profiling and Ultrastructural Analysis Innovative Technologies, Peoples' Friendship University of Russia, Moscow, Russia; f Republican Clinical Perinatal Center, Ufa, Republic of Bashkortostan, 450106, Russia; g Department of Internal Diseases, Bashkir State Medical University, Ufa, Republic of Bashkortostan, 450008, Russia; h Department of Neurosurgery, The First Affiliated Hospital of Harbin Medical University, Harbin, 150001, China; i City Clinical Hospital №21, Ufa, Republic of Bashkortostan, 450071, Russia

**Keywords:** microRNAs, Insulin resistance, Diabetes mellitus, Therapy, Diagnosis, Mechanism

## Abstract

The term "insulin resistance" is commonly understood as a decrease in the response of insulin-sensitive tissues to insulin at its sufficient concentration, leading to chronic compensatory hyperinsulinemia. Type 2 diabetes mellitus is based on mechanisms consisting in the development of resistance to insulin in target cells (hepatocytes, adipocytes, skeletal muscle cells), resulting in the termination of an adequate response of these tissues to interaction with insulin. Since in healthy people 75–80% of glucose is utilized by skeletal muscle, it is more likely that the main cause of insulin resistance is impaired insulin-stimulated glucose utilization by skeletal muscle. With insulin resistance, skeletal muscles do not respond to insulin at its normal concentration, thereby determining an increase in glucose levels and a compensatory increase in insulin production in response to this. Despite many years of studying diabetes mellitus (DM) and insulin resistance, the molecular genetic basis for the development of these pathological conditions is still the subject of numerous studies. Recent studies point to the involvement of microRNAs (miRNAs) as dynamic modifiers in the pathogenesis of various diseases. MiRNAs are a separate class of RNA molecules that play a key role in the post-transcriptional regulation of gene expression. Recent studies have shown that miRNAs dysregulation in DM is closely related to miRNAs regulatory abilities in skeletal muscle insulin resistance. This gave grounds to consider an increase or decrease in the expression of individual microRNAs in muscle tissue and consider them as new biomarkers for diagnosing and monitoring insulin resistance and promising directions for targeted therapy. This review presents the results of scientific studies examining the role of miRNAs in skeletal muscle insulin resistance.

## 1 Introduction

In recent years, the incidence of obesity, type 2 diabetes mellitus (T2DM) and its complications has risen dramatically due to the prevalence of Western-style diets and sedentary lifestyles. Currently, insulin resistance (IR) is believed to be a common pathophysiological mechanism of obesity, metabolic syndrome, T2DM and their complications [1]. IR refers to the physiological effect of a normal insulin concentration below the normal biological response, mainly manifested as a decrease in the sensitivity and reactivity of target tissues to the action of insulin [2]. Skeletal muscle is one of the target organs of insulin action, and 75% of the blood sugar is taken up by it [3]. Therefore, skeletal muscle plays an important role in maintaining the body's glucose homeostasis, and is the earliest and most important part of IR. When IR occurs in skeletal muscle, the levels of free fatty acids and blood sugar increase, which can cause damage to glucose uptake and insulin signaling pathways in skeletal muscle, and can also cause mitochondrial biogenesis and dysfunction in skeletal muscle. In recent years, with the development of molecular biology, a large number of literatures have reported that there are a variety of microRNAs (microRNAs, miRNAs) in eukaryotic cells, which can regulate gene expression and translation at the post-transcriptional level and participate in various life processes. Such as cell proliferation and differentiation, tumors and muscle diseases [4]. Studies have shown that miRNAs play key regulatory roles in IR, T2DM and its complications. In order to explore the relationship between the expression of miRNAs and skeletal muscle IR, this paper systematically combed a large number of domestic and foreign literatures, and explained the regulation of miRNAs on skeletal muscle IR from the aspects of skeletal muscle glucose uptake, insulin signaling pathway, and mitochondrial biosynthesis. This will help to deepen our understanding of the mechanism of miRNAs regulating skeletal muscle IR, provide a new direction for the inhibition or treatment of IR, and provide a reference for medical practice.

## 2 Production of miRNAs and their biological functions

In 1993, Lee et al. discovered miRNA-lin4 in Caenorhabditis elegans [5]. With the development of molecular biology, other scholars successively discovered a variety of miRNAs [6]. miRNAs are 19–22 nucleotides in length and are small compared to other classes of RNA in cells. The generation of miRNAs initially occurs in the nucleus, under the action of RNA polymerase II, the primary transcript pri-miRNA with a hairpin structure is formed [7], and then a stem-loop containing 60–70 nucleotides is formed through the action of Drosha structure of pre-miRNA, and then exportin-5 transports pre-miRNA from the nucleus to the cytoplasm [8], and then undergoes cleavage by endonuclease III Dicer, and the pre-miRNA produces a double-stranded RNA molecule of about 22 nucleotides, One of the single strands forms mature miRNAs, and the other single strand is degraded [8].

Mature miRNAs bind to the 3′ untranslated region (UTR) of target gene mRNA to inhibit protein synthesis [9]. One miRNA can regulate the function of multiple genes, and multiple miRNAs can also regulate a single target gene. MiRNAs have multiple functions, can regulate the expression of 70% of human coding genes, and have a wide range of biological effects in the growth and development of the body, cell proliferation and differentiation, immune system regulation, tumorigenesis, etc. [10]. Recent studies have shown that miRNAs are also involved in the regulation of adipocyte differentiation, glucose and lipid metabolism, and insulin production and secretion, suggesting that miRNAs may be related to the occurrence and development of metabolic diseases such as obesity and IR [11,12].

## 3 New functions of miRNAs - regulation of IR in skeletal muscle

Insulin signaling pathway is the main pathway for glucose uptake in skeletal muscle, and its dysfunction can lead to impaired glucose uptake in skeletal muscle. In addition, mitochondria are organelles for energy production in the body, and decreased mitochondrial content in skeletal muscle is an important pathogenic factor of T2DM [13]. Recent studies have shown that a variety of miRNAs can regulate glucose uptake in skeletal muscle, insulin signaling pathway and mitochondrial biogenesis, thereby participating in the regulation of skeletal muscle IR (Table 1).

**Table 1:** MiRNAs associated with insulin resistance in skeletal muscles.

miRNA	Description	Reference
**miR-29**	Promotes insulin resistance	[14]
**miR-103/107**	Causes insulin resistance by targeting Cav1	[15]
**let-7**	160 patients (tumor tissue vs. adjacent normal tissue)	[16]
**miR-1/133a**	Negatively regulated by insulin through SREBP1c and MEF2C	[17]
**miR-223**	Overexpression increases glucose uptake via inducing Glut4 expression	[18]
**miR-494**	Exacerbates insulin resistance by downregulating Slc2A4	[19]

## 4 Regulation of glucose uptake in skeletal muscle by miRNAs

### 4.1 Decreased skeletal muscle glucose uptake is a manifestation of skeletal muscle IR

Skeletal muscle is the main site of insulin-stimulated glucose uptake, and it is also the multiple site of peripheral IR [20]. Glucose in skeletal muscle is mainly transported by diffusion, which requires the participation of glucose transporter 4 (GLUT4). When there is no insulin stimulation, a large amount of GLUT4 is stored in the tubular structure of GLUT4 storage vesicles (GSVs) [21]. Upon insulin stimulation, GSVs translocate to the cell surface, rapidly release GLUT4, and increase glucose uptake [22]. When IR occurs, GLUT4 translocation is impaired in skeletal muscle, its mRNA and protein expression levels are reduced, plasma glucose uptake is reduced by 55%, and skeletal muscle glucose uptake is reduced by 92% [23,24]. On the contrary, overexpressing GLUT4 specifically in skeletal muscle of diabetic mice can increase muscle glucose uptake and improve blood sugar levels [25]. This suggests that skeletal muscle IR can cause ectopic impairment of GLUT4, leading to decreased glucose uptake in skeletal muscle.

### 4.2 miR-29 and glucose uptake in skeletal muscle

Slc2a4/GLUT4 is a key pathway for the regulation of glucose uptake; studies have shown that various microRNAs are involved in the regulation of this pathway [26]. The miR-29 family includes: miR-29a, miR-29b and miR-29c, all of which regulate GLUT4 expression. Studies have shown that the expression of the miR-29 family is significantly upregulated in the skeletal muscle of obese rodents with IR or diabetes, and overexpression of miR-29a and miR-29c in the mouse tibialis anterior muscle by electroporation can lead to skeletal muscle glucose uptake and glycogen levels decreased, which was accompanied by a decrease in the content of GLUT4 [27]. In addition, reduced expression levels of Slc2a4 mRNA and GLUT4 protein were also observed in C2C12 myoblasts overexpressing miR-29a-3p [27]. The above studies have shown that the miR-29 family may be involved in the regulation of glucose levels in skeletal muscle by regulating the Slc2a4/GLUT4 pathway. It is hypothesized that impaired glucose uptake in skeletal muscle IR may be partially caused by abnormal expression of the miR-29 family.

### 4.3 Other miRNAs and glucose uptake in skeletal muscle

In addition to the miR-29 family, other miRNAs are also involved in the regulation of glucose uptake in skeletal muscle. Studies have shown that in L6 cells overexpressing miR-106b, miR-27a, and miR-30d, both glucose consumption and glucose uptake were reduced, accompanied by down-regulation of GLUT4, MAPK14, and PI3K protein expression [20]. On the contrary, if the expressions of miR-106b, miR-27a and miR-30d in IR-treated L6 cells were inhibited, the protein expression levels of GLUT4, MAPK14 and PI3K increased, and the glucose uptake ability of L6 cells also increased [20]. miR-106b, miR-27a and miR-30d play an important role in glucose uptake and metabolic pathways in skeletal muscle cells, and they may be involved in the occurrence and development of skeletal muscle IR.

However, not all abnormal expressions of miRNAs can inhibit glucose uptake in skeletal muscle. Studies have shown that the expression of miR-24 in skeletal muscle of GK rats is down-regulated, the expression of its targeted p38MAPK is up-regulated, and the transport of GLUT4 is increased, which helps the muscles adapt to higher levels of glucose uptake [28,29]. Similar to miR-24, the expression of miR-126 in IR skeletal muscle is down-regulated, and the activity of its targets p85β and PI3K is increased, which promotes the translocation of GLUT4 to the skeletal muscle cell membrane and increases the uptake of glucose by skeletal muscle cells [30]. Therefore, miR-24 and miR-126 may not be involved in the pathogenesis of skeletal muscle IR, but they may be involved in the adaptation of skeletal muscle cells to elevated glucose levels.

## 5 Regulation of miRNAs on insulin signaling pathway

### 5.1 Impaired insulin signaling is an essential feature of skeletal muscle IR

Insulin binds to its receptor, activates intrinsic kinase activity, and causes insulin receptor substrate 1/2 (IRS1/2) and PI3K phosphorylation, IRS2 is a key factor in insulin signal transduction. Under insulin stimulation, it can interact with SH2 domain-containing PI3K to promote signal transmission. Studies have shown that IRS2-null mice exhibit glucose intolerance and IR, and develop symptoms of hyperglycemia [31]. In addition, studies have shown that the activation of the insulin signal transduction pathway (IRS1/PI3K) in skeletal muscle of T2DM patients is reduced, and the activation of its downstream signaling molecules Akt, PKC-zeta and TBCID4 is impaired, thereby damaging insulin-stimulated vesicles (GSVs) translocation, causing IR in skeletal muscle [32,33]. The above studies have shown that impairment of the insulin signaling pathway in skeletal muscle can cause IR.

### 5.2 MiR-135a and insulin signaling pathway

MiR-135a, a key regulator of myogenesis, targets the 3′UTR of IRS2 mRNA. Studies have shown that the expression level of miR-135a in skeletal muscle of diabetic patients is increased, and higher levels of miR-135a can lead to decreased IRS2 mRNA and protein expression levels, decreased PI3K, p85α and Akt phosphorylation levels, and decreased glucose uptake [31,34]. Inhibiting the expression of miR-135a in C2C12 myoblasts can increase the expression levels of IRS2 and Akt in C2C12 myoblasts, improve glucose tolerance, and reduce the symptoms of hyperglycemia [31]. It is suggested that miR-135a plays a key role in the process of skeletal muscle insulin signal transduction, and its abnormal expression may be the mechanism of impaired skeletal muscle insulin transduction signal.

### 5.3 MiR-1 and insulin signaling pathway

MiR-1 is a kind of miRNAs abundant in skeletal muscle, which is involved in regulating the proliferation and differentiation of skeletal muscle cells, and plays an important role in the physiological and pathological processes of skeletal muscle. Under normal circumstances, miR-1 participates in the insulin signal transduction pathway by regulating the expression of insulin-like growth factor 1 receptor (IGF-1R) and IRS1 [26]. Frias Fde et al. showed that the expression of miR-1 in the soleus muscle of obese mice induced by high-fat diet was significantly reduced, accompanied by a significant reduction in the expression of IGF-1, IRS-1, Rheb and follistatin, and an increase in blood glucose levels, which indicated that miR-1 plays an important role in regulating insulin signal transduction, and its abnormal expression may be an early marker of IR development in skeletal muscle [35].

### 5.4 Let-7 and insulin signaling pathway

Let-7 is one of the first miRNAs found in Caenorhabditis elegans, which can play a role in multiple tissues. In skeletal muscle, it can regulate the insulin signaling pathway by targeting IGF-1R and IRS2, and can also regulate insulin signaling through Acts on PI3K and mTOR pathways to regulate insulin sensitivity and glucose metabolism throughout the body [36]. Studies have shown that transgenic mice overexpressing Let-7 exhibit glucose intolerance and peripheral IR, and knockout of Let-7 in the whole body of mice can reverse the glucose tolerance of diet-induced obese mice. damaged [36,37]. In addition, Let-7 is regulated by lin28a and lin28b, and overexpressing lin28 in mouse skeletal muscle by transgenic method can improve glucose metabolism, which may be related to the decrease of Let-7 expression and IRS2-PI3K-mTOR signaling pathway related to the enhancement [36]. The above findings suggest that Let-7 plays an important role in the insulin signaling pathway, and its abnormal expression may be involved in the occurrence of skeletal muscle IR.

### 5.5 MiR-29 and insulin signaling pathway

Studies have shown that miR-29 can target the 3′UTR of IRS1 mRNA and regulate insulin signaling pathways such as phosphoinositide-3-kinase regulatory subunits 1 and 3 (PIK3R1/3) and Akt2 [9]. Overexpression of miR-29 can lead to the decrease of IRS1, PIK3R3 and Akt2 mRNA expression levels, accompanied by the decrease of IRS1, Akt and GSK3α/β protein phosphorylation levels, indicating that miR-29 plays a role in regulating insulin signal transduction pathway. In addition, the expression of miR-29 was significantly upregulated in IR skeletal muscle or skeletal muscle of obese diabetic rodents, suggesting that the impairment of insulin signal transduction pathway in IR skeletal muscle may be partly caused by abnormal expression of miR-29 [27].

## 6 Regulation of miRNAs on mitochondrial biogenesis in skeletal muscle

### 6.1 Blockage of mitochondrial biogenesis leads to IR in skeletal muscle

Mitochondria are essential organelles in eukaryotes, and their main function is to provide cellular chemical energy in the form of ATP [28]. Any mitochondrial dysfunction may lead to serious metabolic problems. Skeletal muscle mitochondrial oxidative phosphorylation, decreased fatty acid β-oxidative capacity, enhanced oxidative stress, and imbalance of fusion and fission are all associated with skeletal muscle IR [38]. In addition, the reduction of mitochondrial content in skeletal muscle is a pathogenic factor of T2DM, and its content is closely related to mitochondrial biosynthesis [13,39]. Studies have shown that the number of skeletal muscle mitochondria in IR, obese and T2DM animals decreased, their volume decreased, their respiratory capacity decreased, and the expression of mitochondrial biosynthesis-related proteins (such as Tfam, COXIV, Cyt C) decreased, suggesting that: mitochondrial biogenesis disorders are closely related to metabolic disorders and skeletal muscle IR [[40], [41], [42]].

### 6.2 MiR-133a and mitochondrial biosynthesis

MiR-133a is a muscle-rich miRNA with multiple biological functions, including enhancing myoblast proliferation by targeting serum response factors and preventing brown fat deposition in muscle cells by inhibiting PRDM16 [43,44]. In addition, miR-133a is closely related to mitochondrial biosynthesis, and miR-133a-deficient mice have decreased transcription levels of mitochondrial biosynthesis markers PGC-1α, NRF-1 and TFAM in skeletal muscle. It can cause an increase in the expression level of miR-133a, and the expression of mitochondrial biosynthesis markers in skeletal muscle is significantly increased, which indicates that miR-133a plays an important role in mitochondrial biosynthesis in skeletal muscle [45]. Similar to the above animal studies, miR-133a expression was downregulated in skeletal muscle of patients with IR or T2DM, which was accompanied by a decrease in transcript levels of mitochondrial biosynthesis markers in skeletal muscle [46]. These findings suggest that miR-133a may inhibit the transcription of PGC-1α, NRF-1 and TFAM and other factors, leading to the blockage of mitochondrial biogenesis and the occurrence of IR in skeletal muscle.

## 7 MiR-149 and mitochondrial biosynthesis

Mitochondrial biosynthesis is regulated by a variety of factors. Studies have shown that class III histone deacetylase sirtuin-1 (SIRT-1) can directly interact with PGC-1α to regulate mitochondrial biosynthesis [47,48]. However, the activation of PGC-1α by SIRT-1 is completely dependent on the level of free nuclear NAD+, and the level of free nuclear NAD+ is regulated by poly ADP-ribose polymerase 2 (PARP-2). The level of NAD+ in muscle cells increases, and the activity of SIRT-1 and mitochondrial biosynthesis are enhanced [44]. In normal skeletal muscle, miR-149 can inhibit the expression of PARP-2, increase the level of free nuclear NAD+ and the activity of SIRT-1, leading to the activation of PGC-1α and increased mitochondrial biogenesis [49,50]. However, the expression level of miR-149 in skeletal muscle of IR mice induced by high-fat diet decreased, the activation of SIRT-1/PGC-1α pathway decreased, and the mitochondrial biosynthesis markers COX1, Cyt C, estrogen-related receptor α (ERR- α), mitochondrial transcription factor A (mtTFA), nuclear respiratory factor 1/2 (NRF1/2) and UCP1 were all decreased [51]. These results indicated that miR-149 plays an important role in mitochondrial biogenesis of skeletal muscle, and the disorder of mitochondrial biogenesis in skeletal muscle caused by high-fat diet may be partly caused by the abnormal expression of miR-149. Targets of skeletal muscle IR.

## 8 MiR-106b and mitochondrial biosynthesis

MiR-106b is a tumor-associated miRNA, and studies have shown that miR-106b is abnormally expressed in liver cancer, breast cancer, and chronic myelogenous leukemia [[52], [53], [54]]. Recent studies have shown that miR-106b is closely related to skeletal muscle IR and T2DM, and its expression level is increased in skeletal muscle of T2DM patients and 12-week high-fat diet-induced IR mice [46,55]. In addition, miR-106b is also involved in the regulation of mitochondrial biosynthesis. Palmitic acid was used to induce IR in C2C12 myoblasts. The results showed that the expression of miR-106b in C2C12 myoblasts increased, and ATP production and mitochondrial DNA (mtDNA) levels decreased. −106b activity, the level of intracellular reactive oxygen species (ROS) decreases, the expression level of the ERR-α/PGC-1α/Mfn2 axis is up-regulated, and the mitochondrial biosynthesis increases [56]. A similar phenomenon was found in the TNF-α-induced IR model of C2C12 myoblasts [57]. These studies indicate that miR-106b can negatively regulate mitochondrial biogenesis in skeletal muscle, and inhibiting the expression of miR-106b can improve mitochondrial biogenesis and IR. Therefore, miR-106b may be a potential target for the treatment of mitochondrial dysfunction in IR skeletal muscle.

### 8.1 Other miRNAs and mitochondrial biogenesis

In human and mouse muscles, miR-23a can target the 3′UTR of PGC-1α mRNA and regulate the protein expression level of PGC-1α [58], suggesting that miR-23a may be involved in the regulation of mitochondrial biogenesis. Studies have shown that the expression level of miR-23a in skeletal muscle of IR mice is increased, and the protein expression level of PGC-1α is decreased. The expression levels of pigment b and COX IV protein decreased, indicating that miR-23a can negatively regulate mitochondrial biogenesis, and the blockage of mitochondrial biogenesis in skeletal muscle of IR or T2DM individuals may be related to the increase of miR-23a level [58]. In addition, miR-761 can also target the 3′UTR of PGC-1α mRNA. If miR-761 is overexpressed in C2C12 myoblasts by transfection, it can inhibit the p38MAPK/ATF2 signaling pathway, resulting in a decrease in PGC-1α protein level, suggesting that miR-761 can also negatively regulate mitochondrial biogenesis in muscle cells [59].

In summary, the expressions of miR-133a, miR-149, miR-106b, miR-23a and miR-761 in skeletal muscle of individuals with diabetes or IR all have significant changes, and these miRNAs can all regulate mitochondrial biogenesis in skeletal muscle, suggesting that They may have played an important role in the occurrence and development of skeletal muscle IR.

## 9 Conclusion

**Fig. 1:**
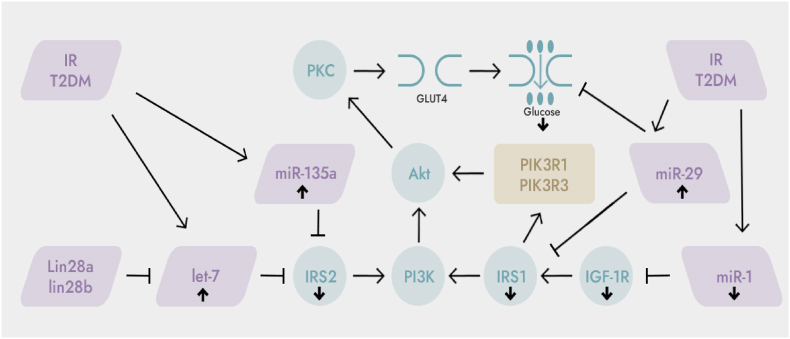
The role of microRNAs in the regulation of the glucose uptake pathway and the insulin signaling pathway. ↑: up-regulation; ↓: down-regulation; IRS1: insulin receptor substrate 1; PI3K: phosphatidylinositol 3-kinase; IRS2: insulin receptor substrate 2; GLUT4: glucose transporter 4. Akt: protein kinase B; PKC: protein kinase C; IGF-1R: insulin-like growth factor-1 receptor; PIK3R1 and PIK3R3: phosphoinositide-3-kinase regulatory subunits 1 and 3; IR/T2DM can up-regulate the expression of Let-7 and miR-135a, thereby inhibiting the expression of its target gene IRS2; IR/T2DM can also up-regulate the expression of miR-29 and inhibit the expression of its target gene IRS1. miR-29a can also directly inhibit glucose uptake. IR/T2DM can down-regulate miR-1 expression, thereby inhibiting the expression of its target gene IGF-1R.

**Fig. 2:**
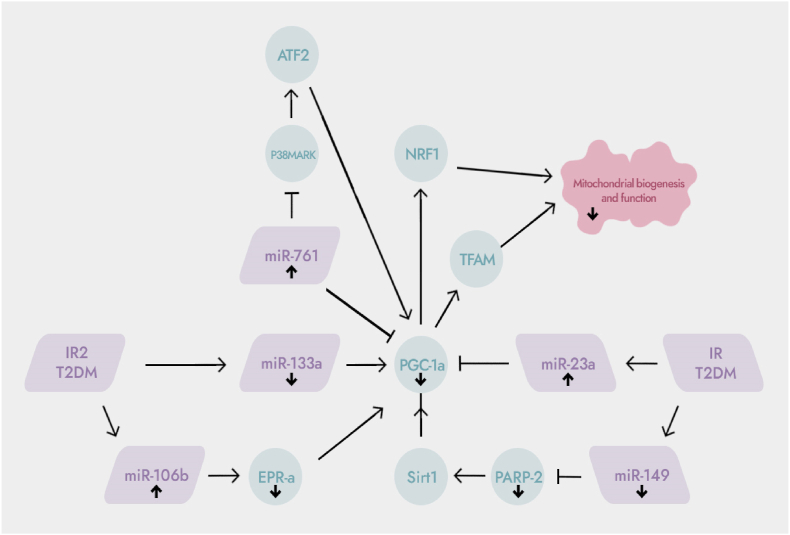
The role of miRNAs in the regulation of mitochondrial biogenesis. ↑: up-regulation; ↓: down-regulation; PGC-1α: peroxisome proliferator-activated receptor coactivator-1α; ERR-α: estrogen receptor-related receptor α; P38MAPK: mitogen-activated protein kinase; NRF1: nuclear respiratory factor 1; TFAM: mitochondrial transcription factor A. PARP-2: poly ADP-ribose polymerase-2; IR or T2DM can resulted in up-regulation of miR-106b expression and down-regulation of miR-149 expression, thereby inhibiting the expression of its target genes ERR-α and PARP-2. IR/T2DM also can up-regulate the expression of miR-761 and miR-23a, and down-regulate the expression of miR-133a, which leads to down-regulation of its target gene PGC-1α.

IR is a common pathophysiological mechanism in the development of obesity and T2DM. Skeletal muscle is one of the main target organs for insulin-mediated glucose uptake, metabolism and utilization, and is the earliest and most important part of IR. Studies have shown that impaired glucose uptake in skeletal muscle, impaired insulin signaling pathway, and impaired mitochondrial biosynthesis are closely related to skeletal muscle IR. When skeletal muscle undergoes IR, the expression of various miRNAs is up-regulated (miR-106b, miR-23a, miR-761, miR-135a, Let-7, miR-29a) or down-regulated (miR-133a, miR-149, miR- 1), they participate in the regulation of skeletal muscle glucose uptake, insulin signaling pathway and mitochondrial biogenesis (Fig. 1, Fig. 2), and play an important role in the occurrence and development of skeletal muscle IR, these miRNAs can be used as therapeutic agents for skeletal muscle IR or potential targets for diabetes.

Although a variety of miRNAs may be involved in the occurrence and development of skeletal muscle IR, the specific mechanism is still unclear and needs further study. In addition, exercise intervention, calorie restriction, and supplementation of antioxidants are important means for the prevention and treatment of IR. Future research can use miRNAs as targets to further study the improvement effects and mechanisms of the above interventions on skeletal muscle IR, and actively explore new methods. This will provide new directions and ideas for the prevention or treatment of IR and diabetes.

## Funding

This work was supported by the Bashkir State Medical University Strategic Academic Leadership Program (PRIORITY-2030).

## Author contributions

Aferin Beilerli, Valentin Kudriashov, Albert Sufianov and Andrey Kostin conceptualized and designed the study. All authors have participated in the acquisition, analysis and interpretation of the data. Sema Begliarzade has drafted the manuscript. Tatiana Ilyasova, Yanchao Liang and Albert Mukhamedzyanov contributed to the critical revisions of the manuscript. Ozal Beylerli supervised the research. All authors agreed on the journal to which the article would be submitted, gave the final approval for the version to be published, and agreed to be accountable for all aspects of the work.

## Declaration of competing interest

The authors declare that no conflicts of interest exist.

## References

[1] Gallagher E.J., Leroith D., Karnieli E. (2010). Insulin resistance in obesity as the underlying cause for the metabolic syndrome. Mt. Sinai J. Med..

[2] Sinaiko A.R., Caprio S. (2012). Insulin resistance. J. Pediatr..

[3] Gareev I., Beylerli O., Aliev G., Pavlov V., Izmailov A., Zhang Y., Liang Y., Yang G. (2020 Aug 20). The role of long non-coding RNAs in intracranial aneurysms and subarachnoid hemorrhage. Life (Basel).

[4] Beylerli O., Gareev I., Pavlov V., Chen X., Zhao S. (2020 May). The role of long noncoding RNAs in the biology of pituitary adenomas. World Neurosurg.

[5] Beylerli O., Gareev I., Sufianov A., Ilyasova T., Guang Y. (2022 Feb 25). Long noncoding RNAs as promising biomarkers in cancer. Noncoding RNA Res.

[6] Gareev I., Gileva Y., Dzidzaria A., Beylerli O., Pavlov V., Agaverdiev M., Mazorov B., Biganyakov I., Vardikyan A., Jin M., Ahmad A. (2021 Aug 26). Long non-coding RNAs in oncourology. Noncoding RNA Res.

[7] Ebert M.S., Sharp P.A. (2012). Roles for microRNAs in conferring robustness to biological processes. Cell.

[8] Borralho P.M., Rodrigues C.M., Steer C.J. (2015). microRNAs in mito- chondria: an unexplored niche. Adv. Exp. Med. Biol..

[9] Beylerli O., Khasanov D., Gareev I., Valitov E., Sokhatskii A., Wang C., Pavlov V., Khasanova G., Ahmad A. (2021 Jun 30). Differential non-coding RNAs expression profiles of invasive and non-invasive pituitary adenomas. Noncoding RNA Res.

[10] Sufianov A., Begliarzade S., Ilyasova T., Liang Y., Beylerli O. (2022 Jul 6). MicroRNAs as prognostic markers and therapeutic targets in gliomas. Noncoding RNA Res.

[11] Sufianov A., Begliarzade S., Ilyasova T., Xu X., Beylerli O. (2022 Sep 22). MicroRNAs as potential diagnostic markers of glial brain tumors. Noncoding RNA Res.

[12] Beilerli A., Begliarzade S., Sufianov A., Ilyasova T., Liang Y., Beylerli O. (2022 Jul 31). Circulating ciRS-7 as a potential non-invasive biomarker for epithelial ovarian cancer: an investigative study. Noncoding RNA Res.

[13] Boushel R., Gnaiger E., Schjerling P., Skovbro M., Kraunsoe R., Dela F. (2007). Patients with type 2 diabetes have normal mitochon- drial function in skeletal muscle. Diabetologia.

[14] Herrera B.M., Lockstone H.E., Taylor J.M., Ria M., Barrett A., Collins S., Kaisaki P., Argoud K., Fernandez C., Travers M.E., Grew J.P., Randall J.C., Gloyn A.L., Gauguier D., McCarthy M.I., Lindgren C.M. (2010 Jun). Global microRNA expression profiles in insulin target tissues in a spontaneous rat model of type 2 diabetes. Diabetologia.

[15] Trajkovski M., Hausser J., Soutschek J., Bhat B., Akin A., Zavolan M., Heim M.H., Stoffel M. (2011 Jun 8). MicroRNAs 103 and 107 regulate insulin sensitivity. Nature.

[16] Frost R.J., Olson E.N. (2011 Dec 27). Control of glucose homeostasis and insulin sensitivity by the Let-7 family of microRNAs. Proc. Natl. Acad. Sci. U. S. A..

[17] Granjon A., Gustin M.P., Rieusset J., Lefai E., Meugnier E., Güller I., Cerutti C., Paultre C., Disse E., Rabasa-Lhoret R., Laville M., Vidal H., Rome S. (2009 Nov). The microRNA signature in response to insulin reveals its implication in the transcriptional action of insulin in human skeletal muscle and the role of a sterol regulatory element-binding protein-1c/myocyte enhancer factor 2C pathway. Diabetes.

[18] Lu H., Buchan R.J., Cook S.A. (2010 Jun 1). MicroRNA-223 regulates Glut4 expression and cardiomyocyte glucose metabolism. Cardiovasc. Res..

[19] Lee H., Jee Y., Hong K., Hwang G.S., Chun K.H. (2013 Dec 11). MicroRNA-494, upregulated by tumor necrosis factor-α, desensitizes insulin effect in C2C12 muscle cells. PLoS One.

[20] Zhou T., Meng X., Che H., Shen N., Xiao D., Song X., Liang M., Fu X., Ju J., Li Y., Xu C., Zhang Y., Wang L. (2016). Regulation of insulin resistance by multiple mirnas via targeting the GLUT4 signalling pathway. Cell. Physiol. Biochem..

[21] Leto D., Saltiel A.R. (2012). Regulation of glucose transport by insulin: traffic control of GLUT4. Nat. Rev. Mol. Cell Biol..

[22] Foley K., Boguslavsky S., Klip A. (2011). Endocytosis, recycling, and regulated exocytosis of glucose transporter 4. Biochemistry.

[23] Correa-Giannella M.L., Machado U.F. (2013). SLC2A4 gene: a prom- ising target for pharmacogenomics of insulin resistance. Pharmacogenomics.

[24] Kim J.K., Zisman A., Fillmore J.J., Peroni O.D., Kotani K., Perret P., Zong H., Dong J., Kahn C.R., Kahn B.B., Shulman G.I. (2001). Glucose toxicity and the development of diabetes in mice with muscle-speciﬁc inactivation of GLUT4. J. Clin. Invest..

[25] Garvey W.T., Maianu L., Hancock J.A., Golichowski A.M., Baron A. (1992). Gene expression of GLUT4 in skeletal muscle from insulin- resistant patients with obesity, IGT, GDM, and NIDDM. Diabetes.

[26] Lima T.I., Araujo H.N., Menezes E.S., Sponton C.H., Araújo M.B., Bomﬁm L.H., Queiroz A.L., Passos M.A., E Sousa T.A., Hirabara S.M., Martins A.R., Sampaio H.C., Rodrigues A., Curi R., Car- neiro E.M., Boschero A.C., Silveira L.R. (2017). Role of microRNAs on the regulation of mitochondrial biogenesis and insulin signaling in skeletal muscle. J. Cell. Physiol..

[27] Massart J., Sjogren R.J.O., Lundell L.S., Mudry J.M., Franck N., O'Gorman D.J., Egan B., Zierath J.R., Krook A. (2017). Altered miR-29 expression in type 2 diabetes influences glucose and lipid metabolism in skeletal muscle. Diabetes.

[28] Kelly D.P., Scarpulla R.C. (2004). Transcriptional regulatory circuits controlling mitochondrial biogenesis and function. Genes Dev..

[29] Ferland-Mccollough D., Ozanne S.E., Siddle K., Willis A.E., Bushell M. (2010). The involvement of microRNAs in type 2 diabetes. Biochem. Soc. Trans..

[30] Guo C., Sah J.F., Beard L., Willson J.K., Markowitz S.D., Guda K. (2008). The noncoding RNA, miR-126, suppresses the growth of neoplastic cells by targeting phosphatidylinositol 3-kinase signaling and is frequently lost in colon cancers. Genes Chromosomes Cancer.

[31] Previs S.F., Withers D.J., Ren J.M., White M.F., Shulman G.I. (2000). Contrasting effects of IRS-1 versus IRS-2 gene disruption on carbohydrate and lipid metabolism in vivo. J. Biol. Chem..

[32] Karlsson H.K., Zierath J.R., Kane S., Krook A., Lienhard G.E., Wallberg-Henriksson H. (2005). Insulin-stimulated phosphorylation of the Akt substrate AS160 is impaired in skeletal muscle of type 2 diabetic subjects. Diabetes.

[33] Vind B.F., Pehmøller C., Treebak J.T., Birk J.B., Hey-Mogensen M., Beck-Nielsen H., Zierath J.R., Wojtaszewski J.F., Højlund K. (2011). Impaired insulin-induced site-specific phosphorylation of TBC1 domain family, member 4 (TBC1D4) in skeletal muscle of type 2 diabetes patients is restored by endurance exercise-training. Diabetologia.

[34] Calderari S., Diawara M.R., Garaud A., Gauguier D. (2017). Biological roles of microRNAs in the control of insulin secretion and action. Physiol. Genom..

[35] Frias Fde T., de Mendonça M., Martins A.R., Gindro A.F., Cogliati B., Curi R., Rodrigues A.C. (2016). MyomiRs as markers of insulin resistance and decreased myogenesis in skeletal muscle of diet-induced obese mice. Front. Endocrinol..

[36] Zhu H., Shyh-Chang N., Segrè A.V., Shinoda G., Shah S.P., Einhorn W.S., Takeuchi A., Engreitz J.M., Hagan J.P., Kharas M.G., Urbach A., Thornton J.E., Triboulet R., Gregory R.I. (2011). DIAGRAM consortium; MAGIC investigators, altshuler D, daley GQ. The Lin28/let-7 axis regulates glucose metabo- lism. Cell.

[37] Deiuliis J.A. (2016). MicroRNAs as regulators of metabolic disease: pathophysiologic signiﬁcance and emerging role as biomarkers and therapeutics. Int. J. Obes..

[38] Sarparanta J., García-Macia M., Singh R. (2017). Autophagy and mitochondria in obesity and type 2 diabetes. Curr. Diabetes Rev..

[39] Picard M., Gentil B.J., McManus M.J., White K., St Louis K., Gartside S.E., Wallace D.C., Turnbull D.M. (2013). Acute exercise remodels mitochondrial membrane interactions in mouse skeletal muscle. J. Appl. Physiol..

[40] Fex M., Nitert M.D., Wierup N., Sundler F., Ling C., Mulder H. (2007). Enhanced mitochondrial metabolism may account for the adaptation to insulin resistance in islets from C57BL/6J mice fed a high-fat diet. Diabetologia.

[41] Morino K., Petersen K.F., Shulman G.I. (2006). Molecular mechanisms of insulin resistance in humans and their potential links with mitochondrial dysfunction. Diabetes.

[42] Phielix E., Schrauwen-Hinderling V.B., Mensink M., Lenaers E., Meex R., Hoeks J., Kooi M.E., Moonen-Kornips E., Sels J.P., Hesselink M.K., Schrauwen P. (2008). Lower intrinsic ADP-stimu- lated mitochondrial respiration underlies in vivo mitochon- drial dysfunction in muscle of male type 2 diabetic patients. Diabetes.

[43] Nielsen S., Scheele C., Yfanti C., Akerström T., Nielsen A.R., Pedersen B.K., Laye M.J. (2010). Muscle speciﬁc microRNAs are reg- ulated by endurance exercise in human skeletal muscle. J. Physiol..

[44] Yin H., Pasut A., Soleimani V.D., Bentzinger C.F., Antoun G., Thorn S., Seale P., Fernando P., van Ijcken W., Grosveld F., Dekemp R.A., Boushel R., Harper M.E., Rudnicki M.A. (2013). MicroRNA-133 controls brown adipose determination in skeletal muscle satellite cells by targeting Prdm16. Cell Metabol..

[45] Nie Y., Sato Y., Wang C., Yue F., Kuang S., Gavin T.P. (2016). Impaired exercise tolerance, mitochondrial biogenesis, and muscle fiber maintenance in miR-133a-deficient mice. Faseb. J..

[46] Gallagher I.J., Scheele C., Keller P., Nielsen A.R., Remenyi J., Fischer C.P., Roder K., Babraj J., Wahlestedt C., Hutvagner G., Pedersen B.K., Timmons J.A. (2010). Integration of microRNA changes in vivo identiﬁes novel molecular features of muscle insulin resistance in type 2 diabetes. Genome Med..

[47] Gerhart-Hines Z., Rodgers J.T., Bare O., Lerin C., Kim S.H., Mostoslavsky R., Alt F.W., Wu Z., Puigserver P. (2007). Metabolic control of muscle mitochondrial function and fatty acid oxidation through SIRT1/PGC-1alpha. EMBO J..

[48] Ryall J.G. (2012). The role of sirtuins in the regulation of metabolic homeostasis in skeletal muscle. Curr Opin Clin Nutr Metab.

[49] Smith J.S., Brachmann C.B., Celic I., Kenna M.A., Muhammad S., Starai V.J., Avalos J.L., Escalante-Semerena J.C., Grubmeyer C., Wolberger C., Boeke J.D. (2000). A phylogenetically conserved NAD+-dependent protein deacetylase activity in the Sir2 protein family. Proc. Natl. Acad. Sci. U. S. A..

[50] Bai P., Cantó C., Oudart H., Brunyánszki A., Cen Y., Thomas C., Yamamoto H., Huber A., Kiss B., Houtkooper R.H., Schoonjans K., Schreiber V., Sauve A.A., Menissier-de Murcia J., Auwerx J. (2011). PARP-1 inhibition increases mitochondrial metabolism through SIRT1 activation. Cell Metabol..

[51] Mohamed J.S., Hajira A., Pardo P.S., Boriek A.M. (2014). MicroRNA- 149 inhibits PARP-2 and promotes mitochondrial biogenesis via SIRT-1/PGC-1alpha network in skeletal muscle. Diabetes.

[52] Li N., Liu Y., Miao Y., Zhao L., Zhou H., Jia L. (2016). MicroRNA- 106b targets FUT6 to promote cell migration, invasion, and proliferation in human breast cancer. IUBMB Life.

[53] Yen C.S., Su Z.R., Lee Y.P., Liu I.T., Yen C.J. (2016). miR-106b promotes cancer progression in hepatitis B virus-associated hepatocel- lular carcinoma. World J. Gastroenterol..

[54] Horie T., Ono K., Nishi H., Iwanaga Y., Nagao K., Kinoshita M., Kuwabara Y., Takanabe R., Hasegawa K., Kita T., Kimura T. (2009). MicroRNA-133 regulates the expression of GLUT4 by tar- geting KLF15 and is involved in metabolic control in cardiac myocytes. Biochem. Biophys. Res. Commun..

[55] Chen G.Q., Lian W.J., Wang G.M., Wang S., Yang Y.Q., Zhao Z.W. (2012). Altered microRNA expression in skeletal muscle results from high-fat diet-induced insulin resistance in mice. Mol. Med. Rep..

[56] Zhang Y., Zhao Y.P., Gao Y.F., Fan Z.M., Liu M.Y., Cai X.Y., Xia Z.K., Gao C.L. (2015). Silencing miR-106b improves palmitic acid- induced mitochondrial dysfunction and insulin resistance in skeletal myocytes. Mol. Med. Rep..

[57] Zhang Y., Yang L., Gao Y.F., Fan Z.M., Cai X.Y., Liu M.Y., Guo X.R., Gao C.L., Xia Z.K. (2013). MicroRNA-106b induces mitochondrial dysfunction and insulin resistance in C2C12 myotubes by targeting mitofusin-2. Mol. Cell. Endocrinol..

[58] Russell A.P., Wada S., Vergani L., Hock M.B., Lamon S., Leger B., Ushida T., Cartoni R., Wadley G.D., Hespel P., Kralli A., Soraru G., Angelini C., Akimoto T. (2013). Disruption of skeletal muscle mitochondrial network enes and miRNAs in amyotrophic lateral sclerosis. Neurobiol. Dis..

[59] Xu Y., Zhao C., Sun X., Liu Z., Zhang J. (2015). MicroRNA-761 regulates mitochondrial biogenesis in mouse skeletal muscle in response to exercise. Biochem. Biophys. Res. Commun..

